# Continuing efforts to integrate care can benefit from cross-jurisdictional comparisons

**DOI:** 10.5334/ijic.2241

**Published:** 2015-09-23

**Authors:** Walter P. Wodchis, Anna Dixon, Geoff M. Anderson

**Affiliations:** IHPME, University of Toronto; Toronto Rehabilitation Institute; Institute for Clinical Evaluative Sciences, Institute of Health Policy Management & Evaluation, Toronto, ON, Canada; Centre for Ageing Better, London, UK; Institute of Health Policy, Management and Evaluation, University of Toronto, Toronto, ON, Canada

Despite its theoretical appeal, integrated care remains a new frontier for health and social care systems in many countries. A key motivation for increasing the integration of a range of health care services with social- or community-based services is the imperative to improve the patient experience, particularly for individuals with ongoing multiple and complex health care and functional needs. To meet these needs they receive care from many different providers and often they feel they are bouncing around in an uncoordinated system that requires them to repeatedly tell their stories but that fails to provide a clearly articulated coordinated plan of care to help them manage their conditions. A key to improving the patient experience, care coordination and outcomes, is the need to address failures associated with low-fidelity of information sharing among providers, as patients transition from one provider to another. Failure to share information on treatment goals and therapies almost inevitably results in an incoherent treatment plan that often is duplicative or self-defeating and that in some cases causes more harm than good. Along with improving the patient experience, integrated care can increase the system-level efficiency of treatment and lower costs. Bringing multiple services into a coordinated network where information is centrally held and shared supports coordinated scheduling, shared access to essential information and reduction in duplication of diagnostic tests. In theory integrated care can improve patient experience and outcomes while reducing costs.

But what does the practice of integrated care mean? Is it simply the smooth flow of information between a primary care and medical specialists? This might be true in patients with clear, simple needs for whom there is a well-defined well-established care plan, for example someone with stable diabetes. But what if the person has not only diabetes, but also hypertension, mild renal impairment, arthritis that limits mobility, mild depression and moderate cognitive impairment. This means a much larger array of relevant providers and a complexity of needs that is not easy to fit into a well-defined and well-established care plan. This type of individual is best served by care goals and care plans that are uniquely tailored and based on clear communication among providers and patients and their caregivers. In the context of multiple chronic conditions or a mix of medical and functional/personal support needs, a broader set of services need to be included within a single integrated care bundle. Indeed different types and intensities of services available within integrated care programmes are appropriate for people with different types of care needs. In order to be cost-effective, it is essential that the specific programme services be well matched to the needs of the patient. The optimal model is patient-centred integrated services, for which organizational integration may not be necessary nor sufficient.

The global increase in the prevalence of chronic disease and particularly the growing number of individuals with multiple chronic conditions is increasing the emphasis and attention on integrated care programmes internationally. In all modern health and social care systems, the relatively small proportion of the population (generally less than 10%) with multiple functional and medical challenges, many of whom have multiple chronic conditions, consume a majority of care resources (generally more than 2/3). This creates a real opportunity to learn from programmes that operate in different jurisdictions to manage what are essentially common core challenges of information exchange, treatment planning, coordinated funding and delivery.

This issue of the *International Journal of Integrated Care* examines the practice of integrated care in countries that have many different forms of insurance, payment and delivery systems. They are all facing the same challenge in terms of providing integrated care and we have selected specific interventions and programmes that stand out in each country. Certainly, the implementation or process of integrating care will depend upon the existing organization of patient and information flow across providers as well as the extent of providers who need to be effectively engaged in order to deliver integrated care to a target group of patients. In this regard context becomes critical. The model and the process of integrating care in one locality may need adaptation to be implemented in another jurisdiction. As Toni Ashton mentions in her perspectives paper in this issue, a greater appreciation of best practice for the successful implementation of integrated care is needed. A review of world-leading programmes such as those highlighted in this issue is meant to bring to light some of the key enablers and barriers to the successful implementation of integrated care programmes.

The goals for this issue are to understand the practice of integrated care from an international perspective and to learn from such exemplar initiatives to inform replication and spread. What types of programmes have been introduced around the world and what issues and challenges they were designed to address? What are some essential learning for providers and local agencies trying to implement these programmes? What can payers and regulators/policy makers do to encourage appropriate development and delivery of integrated care? Can integrated care programmes provide better care and experience, lead to better outcomes and lower cost?

This issue suggests that there are important components and approaches that programmes can adapt and implement. It also suggests that sustained support from senior decision-makers over many years is necessary for programmes to reach scale and sustained implementation. At the front lines, physician engagement is needed to improve the integration of medical and social care. Programmes have found greater success by focusing on integrating services at the front lines and supporting patients and their caregivers to become more pro-active in the management of their own care. Finally, there is clearly a continuing need for more robust evaluation supported by sensitive, valid and reliable outcome measures.

